# Effects of HIV-Related Discrimination on Psychosocial Syndemics and Sexual Risk Behavior among People Living with HIV

**DOI:** 10.3390/ijerph17061924

**Published:** 2020-03-16

**Authors:** Randolph C. H. Chan, Don Operario, Winnie W. S. Mak

**Affiliations:** 1 Department of Special Education and Counselling, The Education University of Hong Kong, Hong Kong 999077, China; rchchan@eduhk.hk; 2 Department of Behavioral and Social Sciences, School of Public Health, Brown University, Providence, RI 02912, USA; don_operario@brown.edu; 3 Department of Psychology, The Chinese University of Hong Kong, Hong Kong 999077, China

**Keywords:** stigma, discrimination, psychosocial syndemics, condom use, people living with HIV

## Abstract

In the context of HIV-related stigma and discrimination, people living with HIV (PLHIV) might be vulnerable to a ‘syndemic’ of co-occurring psychosocial challenges that can affect sexual behavior. The present study examined how HIV-related discrimination contributes to co-occurring psychosocial syndemic problems and results in inconsistent condom use among PLHIV in Hong Kong. Two-hundred and ninety-one PLHIV were recruited to complete a self-report questionnaire. More than one-quarter of the sample experienced two or more psychosocial syndemic problems, and 74.1% of the participants who had sex with steady partners reported inconsistent condom use over the past three months. The results indicated that HIV-related discrimination was positively predictive of the number of psychosocial syndemic problems. HIV-related discrimination and psychosocial syndemics were associated with increased odds of inconsistent condom use with steady partners (AOR = 5.40 and AOR = 3.09 respectively). Findings from structural equation modeling showed that psychosocial syndemics mediated the effect of HIV-related discrimination on condom use consistency with steady partners. PLHIV in Hong Kong suffered from the syndemic effects of stigma, social isolation, and poor mental health, which rendered them vulnerable to condomless sex. In order to curb the rapidly increasing incidence of HIV, multi-level strategies should be adopted to concurrently address the structural inequities and psychosocial syndemics faced by PLHIV.

## 1. Introduction

Despite the increasing knowledge of the prevention and control of HIV infections, HIV remains a highly stigmatized condition in most parts of the world. HIV-related discrimination is so rampant and pervasive that people living with HIV (PLHIV) often face rejection and unequal treatment in multiple domains of life, including family and social networks [1], work and educational settings [2], as well as healthcare and social service settings [3]. A global survey study of 2035 PLHIV showed that 78% of the respondents have experienced some forms of stigma and discrimination based on their HIV status [4].

Discrimination toward PLHIV is linked to poor mental health outcomes, including lower self-esteem [5], psychological distress [6], depression [7], post-traumatic stress [8], and suicidal attempts [9]. PLHIV who face discrimination often internalize negative societal stereotypes and develop self-stigma toward their identity [10]. They are likely to disengage from social interaction and withdraw from their community, resulting in social isolation [11,12].

Apart from the adverse effect on psychosocial outcomes, previous studies also found that discrimination may render PLHIV more vulnerable to sexual risk behavior, including condomless sex, which potentially accelerates the spread of the HIV epidemic [5]. Using a national sample of PLHIV in France, Peretti-Watel and colleagues [13] found that the experiences of discrimination from relatives, friends, and colleagues were related to an increase in condomless sex. Paz-Bailey et al. [14] also showed that discrimination was positively related to condomless sex among PLHIV in Honduras. Although these findings supported the association between discrimination and sexual risk behavior in PLHIV, very few have offered theoretical explanations for the relationship.

### 1.1. Syndemic Theory

A syndemic is a constellation of closely intertwined behavioral and psychosocial problems that accelerate health risks in vulnerable populations [15]. It was first introduced in the context of HIV/AIDS by Singer [16], to describe the co-occurring epidemics of substance use, violence, and HIV risk faced by urban minority populations in the United States. Syndemic theory posits that co-occurring psychosocial problems interact and reinforce one another, which disproportionately burden vulnerable populations and ultimately create a snowballing effect on their overall health [17].

Studies applying the syndemic theory have mostly examined how multiple psychosocial problems work additively to increase the vulnerability to HIV infection in marginalized populations [18]. Research has identified the co-occurrence of different psychosocial problems, including poor mental health, substance use, self-stigma, violence, and social isolation, in HIV-vulnerable populations [19,20]. These psychosocial problems have been consistently found to be interrelated with one another, synergistically enhancing the risk of condomless sex and HIV infection among women [19], racial minorities [20], immigrant workers [21], transgender individuals [22], sex workers [23], and men who have sex with men (MSM) [17,24].

The syndemic theory also highlights that co-occurring psychosocial conditions are perpetuated because of deleterious social conditions [15]. It points to social marginalization and victimization as components of an adverse structural environment, through which psychosocial problems emerge and concentrate. A cluster of syndemic conditions may develop as a result of negative life experiences including discrimination. Research by Operario et al. [22] supported these claims, by showing that stigma and discriminatory experiences were positively associated with mutually reinforcing syndemic conditions among transgender women. Coulter et al. [25] found that sexual minority women who encountered sexual orientation-based discrimination were more likely to report co-occurring problems of depression, substance use, and sexual risk behavior. In a sample of young MSM, Herrick et al. [26] further showed that adverse life events were related to increased HIV risk taking, through the production of multiple syndemic problems.

Although existing research evidence supported the syndemic effect of co-occurring psychosocial problems on sexual risk behavior, most of the studies were conducted in populations at risk for HIV infection. Less attention has been paid to understanding the psychosocial syndemics of PLHIV, which might increase their likelihood of engaging in condomless sexual intercourse and compound the risk of HIV transmission [27]. Drawing on the syndemic theory, we propose psychosocial syndemics as a mediating mechanism underlying HIV-related discrimination and sexual risk behavior among PLHIV. We hypothesize that PLHIV who experienced discrimination would report a higher level of co-occurring psychosocial problems, which in turn would contribute to engagement in condomless sex.

### 1.2. HIV and Discrimination in Hong Kong

The HIV epidemic is considered one of the significant public health challenges in Hong Kong. Since the first diagnosis of the HIV infection in 1984, there has been a steady increase in Hong Kong’s HIV infection rate, with a rapid rise documented in the past few years. A recently released government report showed that the HIV incidence in 2017 has hit its highest level since 1984 [28]. It is projected that the number of PLHIV in Hong Kong will surge by 35% in the coming four years [29]. Sexual contact has been the major route of HIV transmission over the past two decades, and there has been a shift in the trend of HIV transmission from heterosexual contact to male-to-male sexual contact in the past ten years. A population-based study revealed that only 42.9% of MSM in Hong Kong reported consistent condom use [30]. However, limited studies have examined the determinants of condom use inconsistency among PLHIV in Hong Kong.

In Hong Kong, PLHIV are legally protected from discrimination under the Disability Discrimination Ordinance [31], which pertains to any social category that can be targeted for discrimination. It is unlawful to subject someone (including PLHIV and their associates) to discrimination, harassment, and vilification on the ground of their HIV status. Despite legal protection, discrimination toward PLHIV is still highly pronounced in Hong Kong [32]. Lau and colleagues [33] found that over 50% of PLHIV in Hong Kong experienced daily discrimination in different settings, including the workplace and social relationships. In a random household survey commissioned by the Equal Opportunities Commission in Hong Kong [34], nearly one-third of the public respondents stated they would avoid contact with PLHIV (30%) and did not want PLHIV to live in their neighborhood (34%), and only a small proportion of the respondents would accept PLHIV as casual friends (22%) or fellow employees (10%). The prevalence of stigma can be attributed to the negative connotations associated with HIV (e.g., sexual immorality, promiscuity, and contagiousness) in the Chinese culture [35].

In accordance with the syndemic theory, prevalent discrimination toward PLHIV may partially account for the psychological distress and behavioral risk factors that contribute to the growth of the HIV epidemic in Hong Kong [36]. The theory provides a framework for illustrating how adverse social conditions contribute to the production of psychosocial syndemics and sexual risk behavior [15]. In light of the lack of understanding toward HIV-related discrimination and co-occurring psychosocial problems in the syndemics literature, as well as the paucity of research on sexual risk behavior among PLHIV in Hong Kong, it is both theoretically and empirically important to elucidate the process and mechanism underlying HIV-related discrimination and sexual risk behavior among PLHIV.

### 1.3. The Present Study

Grounded on the syndemic theory, the present study aimed to (1) assess the co-occurrence of psychosocial syndemics among PLHIV in Hong Kong, (2) investigate the impact of HIV-related discrimination on psychosocial syndemics, (3) understand the effect of HIV-related discrimination and psychosocial syndemics on inconsistent condom use, and (4) examine the mediating effect of psychosocial syndemics on the relationship between HIV-related discrimination and inconsistent condom use.

## 2. Methods

### 2.1. Participants

A total of 303 people living with HIV were screened and determined to be eligible, and consented to participate in the study. The inclusion criteria were (1) being 18 years of age or above, (2) having been diagnosed with HIV infection, and (3) residing in Hong Kong. Of these, 12 participants withdrew from the study due to insufficient time to complete the questionnaire, yielding a final sample of 291 participants. The majority of the participants were male (96.1%, *n* = 268) and ethnically Chinese (94.6%, *n* = 262), with a mean age of 41.82 years (SD = 11.15). Participants were mostly single (67.7%, *n* = 189), and nearly half completed tertiary education (46.4%, *n* = 129). Most were employed full-time (63.9%, *n* = 179). Their mean years since HIV diagnosis was 5.25 years (SD = 4.70). Table 1 describes the demographic characteristics of the participants.

### 2.2. Procedure

The study was approved by the clinical research ethics committees of the corresponding author’s institution and the Department of Health of the Hong Kong Government. Participants were recruited and screened by the nurses at the only community-based HIV clinic in Hong Kong from January to July 2013. Eligible participants were referred to the on-site research staff member, who explained the study background and obtained informed consent from the participants, emphasizing that participation was completely voluntary and their participation would not affect their services at the clinic, participants could withdraw from participation at any time, and all data would be collected anonymously. After providing consent, participants completed a self-report questionnaire in either Chinese or English. It took approximately 40 min to complete the questionnaire. Assistance in reading, interpreting, or filling out the questionnaire was provided upon the request of the participants. They received HK$60 (U.S.$7.70) as compensation for their time spent completing the questionnaire. Participants who felt distressed following the study were advised to seek help from the clinical social workers at the clinic.

### 2.3. Measures

HIV-related discrimination. Participants were asked to indicate how often they had experienced avoidance and/or discrimination based on their HIV-status from different persons in their family and social network (i.e., parents, children, spouse/partner, siblings, other relatives, sex partners, and friends), work and educational settings (i.e., colleagues, supervisors, fellow students, and teachers), as well as healthcare service settings (i.e., HIV-specific healthcare workers and general healthcare workers at general out-patient clinics, accident and emergency units, specialist out-patient clinics, private medical clinics, and private hospitals) over the past year [37]. It consists of 17 items, with each item representing one of the parties stated above. The participants rated each item on a 5-point scale from 0 (never) to 4 (all of the time). A dichotomous variable was computed for data analysis. If the participants indicated a non-zero response (1–4) on one or more of the 17 items, they would be considered as having experience of HIV-related discrimination over the past year.

Psychosocial syndemics. Psychosocial syndemics were indicated by four psychosocial problems (i.e., depression, anxiety, social isolation, and self-stigma). The 4-item depression subscale of the Mental Health Inventory (MHI-18) [38] was used to assess the severity of depression on a 6-point Likert scale from 1 (none of the time) to 6 (all of the time). A sample item includes “How much of the time did you feel depressed?” Cronbach’s alpha in this study was 0.86.

The 5-item anxiety subscale of the Mental Health Inventory (MHI-18) [38] was used to assess the severity of anxiety on a 6-point Likert scale from 1 (none of the time) to 6 (all of the time). A sample item includes “How much of the time have you felt tense or high-strung?” Cronbach’s alpha in this study was 0.80.

For social isolation, the family and friends subscales of the Multidimensional Scale of Perceived Social Support (MSPSS) [39] were used to assess feeling of social connection with family members and friends on a 7-point Likert scale from 1 (very strongly disagree) to 7 (very strongly agree). A sample item includes “I have friends with whom I can share my joys and sorrows.” Cronbach’s alpha in this study was 0.88. The items were reversely coded and averaged to reflect the level of social isolation.

The 9-item Self-Stigma Scale (SSS) [10] was used to evaluate the extent of internalized stigmatizing beliefs about their identity as a person living with HIV on a 6-point Likert scale from 1 (strongly disagree) to 6 (strongly agree). A sample item is “The identity of being a person living with HIV taints my life.” Cronbach’s alpha in this study was 0.91.

We created a composite syndemic index, consistent with the procedures used in previous studies that have adopted the syndemic approach [40,41]. First, the rating for the items of each scale was averaged, to produce a mean score for each psychosocial variable. Second, the midpoint of the scale was used to dichotomize each psychosocial variable, i.e., depression (midpoint = 3.5), anxiety (midpoint = 3.5), social isolation (midpoint = 4), and self-stigma (midpoint = 3.5). For instance, participants with depression scores higher than 3.5 were considered as having higher levels of depression (i.e., coded as 1), whereas participants with depression scores lower than or equal to 3.5 were considered as having lower levels of depression (i.e., coded as 0). Finally, we created the composite syndemic index by summing up dichotomous indicators of high versus low scores (i.e., 1 versus 0) on the four psychosocial problems. The syndemic index ranged from 0 to 4, to indicate the number of psychosocial conditions in each participant, with 0 representing no co-occurring psychosocial problems and 4 representing four co-occurring psychosocial problems.

Sexual risk behavior. Participants reported whether they had a steady and/or casual partner(s) and on the frequency of having sex with these partners over the past three months. A steady partner is a person to whom one is committed in an ongoing dating relationship. Examples of a steady partner (i.e., boyfriend/girlfriend and husband/wife) were provided to the participants. A casual partner is a person with whom one has sex, but does not show emotional attachment. For participants who indicated they have had sex with steady and/or casual partners, they were asked to report the frequency of condom use when having vaginal, anal, and oral sex with steady and/or casual partners over the past three months on a 5-point scale, ranging from 0 (none of the time), 1 (a little bit of the time), 2 (sometimes), 3 (most of the time), and 4 (every time). The responses were then recoded into two variables (i.e., inconsistent condom use with steady partners and inconsistent condom use with casual partners). Participants who reported 0–3 were considered as having inconsistent condom use (i.e., coded as 1), whereas those who reported 4 (i.e., using condom every time) were considered as having consistent condom use (i.e., coded as 0).

### 2.4. Data Analysis

Descriptive statistics were conducted for HIV-related discrimination, psychosocial syndemics, and sexual risk behavior. Independent samples *t*-tests were conducted to examine the difference in the level of psychosocial syndemic problems between participants who had experienced HIV-related discrimination and those who did not experience HIV-related discrimination. Cohen’s *d* was used to determine the degree of difference, whereby a value of 0.02 indicates a small effect size, 0.50 indicates a medium effect size, and 0.80 indicates a large effect size [42]. Pearson correlation coefficients were used to determine the association between different psychosocial syndemic problems.

To investigate the effect of HIV-related discrimination on psychosocial syndemic problems, we conducted Poisson regression analysis, adjusting for demographics (i.e., gender, age, ethnicity, education, employment status, marital status, sexual orientation, length of being diagnosed with HIV infection, and use of antiretroviral therapy). Poisson regression was used because the outcome variable was the number of psychosocial syndemic problems (from 0 to 4). The variable was count data in nature and followed the Poisson distribution. Demographic variables were dummy coded for the analysis.

Logistic regression was conducted to examine the effect of HIV-related discrimination and psychosocial syndemics on inconsistent condom use with steady and casual partners, controlling for demographics. Logistic regression was used because of the dichotomous nature of the outcome variables (i.e., consistent versus inconsistent condom use).

Structural equation modeling was then conducted to examine the mediating effect of psychosocial syndemics on the relationship between HIV-related discrimination and inconsistent condom use. In the model, the latent variable of psychosocial syndemics was indicated by the mean score of depression, anxiety, social isolation, and self-stigma. Weighted least-squares means and variance-adjusted (WLSMV) estimation was used, because it is robust in analyzing both continuous and dichotomous outcome variables [43]. Associations were reported as linear regression coefficients for the continuous outcome variable (i.e., psychosocial syndemics) and probit regression coefficients for the dichotomous outcome variable (i.e., inconsistent condom use). Five indices were employed to evaluate goodness-of-fit for the models, including chi-square (χ^2^) statistics, comparative fit index (CFI), Tucker–Lewis index (TLI), the root mean square error of approximation (RMSEA), and the weighted root-mean-square residual (WRMR). The non-significance of χ^2^ values is an indication of a good model [44]. CFI and TLI values above 0.90 are considered as an acceptable model fit, whereas a RMSEA value of 0.06 or less indicates a close model fit [44]. A WRMR value of 1.00 or less is interpreted as acceptable model fit [45].

The indirect effect of HIV-related discrimination on inconsistent condom use was estimated using a bootstrapping procedure. Bias-corrected 95% confidence intervals (CIs) were generated, based on 1000 bootstrap samples. An indirect effect is interpreted as significant if the 95% CIs do not contain zero. Analyses were conducted using SPSS version 25.0 (IBM Corporation, Armonk, NY, USA) and Mplus version 7.1 (Muthén & Muthén, Los Angeles, CA, USA).

## 3. Results

Prior to the main analyses, descriptive statistics, including means, standard deviations, skewness, and kurtosis, were conducted (see Table 2). An examination of skewness and kurtosis estimates indicated that the values ranged from −0.34 to 0.79 for skewness, and from −0.53 to 1.34 for kurtosis. The skewness and kurtosis fell with the acceptable ranges for normally distributed variables [46].

### 3.1. HIV-Related Discrimination and Psychosocial Syndemics

Approximately one-third of PLHIV (32.3%, *n* = 94) reported experience of HIV-related discrimination over the past one year. More than one-quarter of them (27.1%, *n* = 79) experienced two or more problems. As reported in Table 2, results showed positive, moderate correlations between depression, anxiety, social isolation, and self-stigma (*rs* ranged from 0.30 to 0.76). Comparing psychosocial syndemic problems by HIV-related discrimination, PLHIV who reported HIV-related discrimination reported higher levels of depression (*t* = 3.81, *p* < 0.01, Cohen’s *d* = 0.47), anxiety (*t* = 2.50, *p* = 0.01, Cohen’s *d* = 0.32), and social isolation (*t* = 3.39, *p* < 0.01, Cohen’s *d* = 0.42) than those who did not report HIV-related discrimination.

Table 2 presents the results of the Poisson regression model predicting psychosocial syndemics. HIV-related discrimination was significantly associated with greater levels of psychosocial syndemics (IRR = 1.47, 95% CI 1.14–1.89), after controlling for gender, age, ethnicity, education, employment status, marital status, sexual orientation, length of being diagnosed with HIV infection, and use of antiretroviral therapy.

### 3.2. Condom Use with Steady and Casual Partners

More than one-third of the participants engaged in sexual behavior with their steady (37.1%, *n* = 108) and casual partners (36.8%, *n* = 107) over the past three months. Among those who have had sex with steady partners, only one-fourth of them (25.9%) reported using condoms consistently in every sexual episode. For those who engaged in sex behavior with casual partners, less than one-fifth (18.7%) reported consistent condom use.

Logistic regression was conducted to examine the effect of HIV-related discrimination and psychosocial syndemics on inconsistent condom use with steady and casual partners, adjusting for demographics. As shown in Table 3, HIV-related discrimination was positively related to inconsistent condom use with steady partners (AOR = 5.40, 95% CI 1.09–26.70). Psychosocial syndemics were also associated with the increased odds of inconsistent condom use with steady partners (AOR = 3.09, 95% CI 1.22–7.82). HIV-related discrimination and psychosocial syndemics were not related to condom use with casual partners.

### 3.3. A Mediation Model of HIV-Related Discrimination on Sexual Risk Behavior

**Figure 1:**
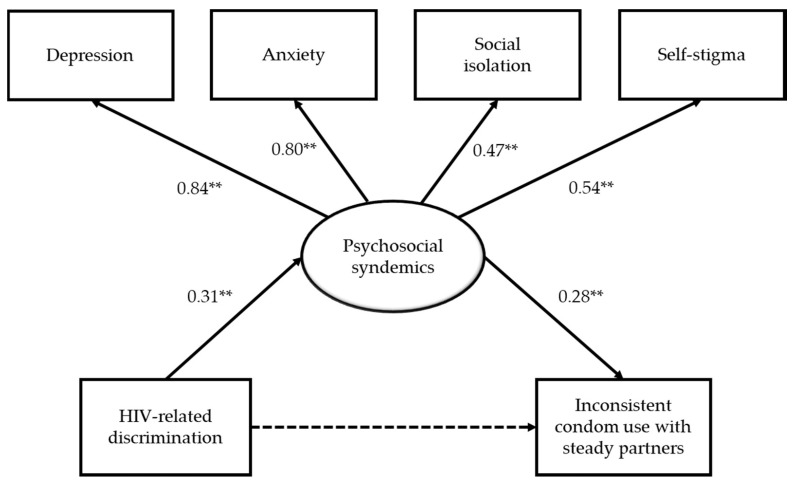
A mediation model of HIV-related discrimination on sexual risk behavior. Notes. Coefficients are standardized linear regression coefficients for psychosocial syndemics and standardized probit regression coefficients for inconsistent condom use; Controlling for gender, age, ethnicity, education, employment status, marital status, sexual orientation, length of being diagnosed with HIV infection, and use of antiretroviral therapy; ** *p* < 0.01.

Structural equation modeling was conducted to examine the hypothesized relationship between HIV-related discrimination, psychosocial syndemics, and inconsistent condom use with steady partners (see Figure 1). Results showed a good model fit, *χ^2^* = 63.16 (*df* = 48, *p* = 0.07), CFI = 0.93, TLI = 0.90, RMSEA = 0.04, WRMR = 0.90. After controlling for demographics, HIV-related discrimination was positively related to psychosocial syndemics (*β* = 0.31, *p* < 0.01), but was not directly related to inconsistent condom use with steady partners (*β* = 0.21, *p* > 0.05). Psychosocial syndemics was positively associated with inconsistent condom use with steady partners (*β* = 0.28, *p* < 0.01). The results also indicated that the indirect effect of HIV-related discrimination on inconsistent condom use via psychosocial syndemics was significant (B = 0.26, 95% CI = 0.02, 0.68). The model explained 56.9% of the variance in the inconsistent condom use with steady partners.

## 4. Discussion

This is one of the first known studies to examine discrimination and the syndemic of adverse psychosocial conditions that affect PLHIV in Hong Kong. One-third of the sample reported experiencing discrimination based on their HIV status during the past year, and one-third also reported two or more adverse psychosocial conditions, which indicated the presence of a psychosocial syndemic in this population. Co-occurring self-stigma and social isolation were the more commonly reported psychosocial syndemic conditions. In addition, our data raises a significant public health concern that inconsistent condom use is highly prevalent among PLHIV in Hong Kong. Three quarters of the sexually active PLHIV reported inconsistent condom use with steady partners, and more than four-fifths of them showed inconsistent condom use with casual partners, which was much higher than the prevalence of condom use inconsistency found in MSM [30] and sex workers [47] in Hong Kong.

The present study is also one of the few studies to demonstrate that psychosocial syndemics among PLHIV can contribute to condomless sex and onward risk for HIV transmission. The findings were consistent with a recent study by van den Berg et al. [27], who found that the syndemic effects of substance use, emotional distress, and social isolation contribute to condomless sex among youth living with HIV in the United States. The present study extends this line of research by recognizing HIV-related discrimination as a key determinant of psychosocial syndemics and inconsistent condom use among PLHIV. Regression analyses and structural equation models supported the hypothesis that discrimination was associated with condomless sex with steady partners, and that this association was mediated through the presence of psychosocial syndemic conditions among PLHIV. In accordance with the syndemic theory, findings highlight that HIV transmission risk in Hong Kong is influenced by a context of HIV-related discrimination toward PLHIV, which affects mental well-being and behavioral risk factors. Efforts to reduce the growing burden of HIV in Hong Kong must therefore consider the structural factors that marginalize PLHIV and address the psychosocial conditions that determine behavioral transmission.

It is also worth noting that the relationship between HIV-related discrimination and condom use consistency differed with respect to condom use with steady partners or casual partners. In contrast to the results shown in steady partners, a non-significant association between HIV-related discrimination and inconsistent condom use with casual partners was observed. This is in line with the proposition by Starks et al. [48], that different factors are involved in instances of unprotected sexual intercourse depending on whether the sexual activity is with a steady partner or a casual partner. There are some additional factors that need to be considered in understanding inconsistent condom use within the context of casual sex encounters. First, condom use self-efficacy in PLHIV may vary across partner types [49]. Given the pervasiveness of HIV stigma, PLHIV might be fearful of disclosing their HIV status, resulting in limited negotiation power over condom use, especially when interacting with casual partners [50]. This is also evident in the present study, where there was a considerably higher rate of inconsistent condom use with casual partners than with steady partners. Second, as the persons involved in casual sex encounters differ from time to time, the dynamics of condom use negotiation with different casual partners can vary substantially. Therefore, there are complex mechanisms of power and agency in shaping condom use dynamics with casual partners, which were not captured in our study. Future research and intervention on HIV prevention should always take into account the relational context of sexual risk behavior [51].

### 4.1. Relevance to Local Context

Hong Kong presents a compelling scenario for examining these issues. Unlike many parts of the world, the HIV epidemic in Hong Kong is relatively young. After the first HIV case in Hong Kong was identified in 1984, the epidemic remained relatively steady, until recent years, when a spike in new infections was observed [28]. Due to this escalating epidemic and the projected upsurge in HIV incidence [29], Hong Kong has not yet developed a robust infrastructure for broad HIV education and awareness, which results in the limited understanding of HIV transmission and social awareness about PLHIV. Moreover, public attitudes in Hong Kong remain influenced by traditional values related to modesty, privacy, and sex, and generally contribute to the aversion of topics related to sexuality and sexual health [52]. Notably, recent studies have documented that even health care providers in Hong Kong evince stigmatizing attitudes and experience discomfort discussing matters related to sexual health with patients [53,54], which highlights the potential exposure of PLHIV to discrimination in health service settings in Hong Kong.

This study links the high prevalence of inconsistent condom use to the widespread HIV stigma in Hong Kong [33,34], showing that discrimination may precipitate co-occurring psychosocial conditions among PLHIV, which in turn predispose them to greater risk of condomless sex. Given such results, current biomedical and behavioral approaches for HIV prevention are likely to be inadequate in the absence of efforts to address negative social judgment toward PLHIV, misunderstanding about HIV, and internalized stigma among PLHIV in Hong Kong [55].

The findings should also be interpreted with an intersectional lens [56]. As evidenced in the present study, a substantial proportion of PLHIV in Hong Kong were sexual minorities. Apart from HIV-related stigma, they face minority stress stemming from the stigma surrounding their same-sex attraction and behavior [57]. It is partly due to the fact that sexual minorities are not entitled to legal protection against sexual orientation-based discrimination in Hong Kong. As a result of their intersectional minority status, HIV-positive gay and bisexual men are particularly vulnerable to psychosocial syndemics [58]. Having multiple marginalized identities might also place them at a disproportionate risk for onward HIV transmission [59]. In order to reduce HIV incidence, psychosocial interventions should target this population and address the syndemic problems that interfere with their safer sex practices.

### 4.2. Strengths and Limitations

One of the key strengths of this paper was in the use of two complementary statistical analyses to examine the theory-based syndemic processes contributing to condomless sex among PLHIV in Hong Kong. Most existing syndemic analyses have applied an additive approach, to understand the cumulative effect of psychosocial syndemics, assuming individual psychosocial conditions contribute equally to sexual health risks in vulnerable populations [60]. To address this limitation, the present study complemented the additive analysis used previously, with structural equation modeling, to model a latent syndemic variable by estimating the relationships between different psychosocial syndemic indicators and weight their relative contributions to the underlying construct [61]. Additional strengths of the study include the large sample of PLHIV in Hong Kong (among the first and largest known studies to date) and the use of validated measures to assess hypothesized relationships.

There are important limitations of this study. First, due to the cross-sectional design, we cannot determine causal relationships or temporal ordering among the variables. As the syndemic theory posits that adverse events across the life span may perpetuate co-occurring psychosocial problems, future studies using prospective designs and assessing social adversity (including discrimination) at multiple points in time will be useful for understanding how early exposure to adverse life experiences contributes to psychosocial syndemics and sexual risk behavior. Second, the use of non-representative sampling restricts the generalizability of findings to the PLHIV population in Hong Kong, particularly those who are not engaged in HIV care services. The sample was also predominantly male, and thus the results might not be applicable to women living with HIV. Third, although all the measures have been validated and used in the Chinese population [10,62,63], they do not have clinical cutoff scores to indicate the presence or absence of the psychosocial syndemic problems. Future works should adopt measures with well-established and validated cutoff values to differentiate individuals with clinically significant levels of problems. Fourth, wide confidence intervals were observed in results of some of the regression analyses, indicating the uncertainty of effect sizes, that was linked with low counts for observed variables. Fifth, while this study attempted to understand the role of structural and psychosocial factors in shaping condom use behavior, we recognized that condom use is also determined by other behavioral, relational, and contextual factors (e.g., condom use self-efficacy, condom negotiation skills, condom availability, alcohol use) that were not investigated in the present study [64,65]. Other confounding factors, such as the HIV status of the participants’ partners, were not measured, and thus we did not know the extent to which serosorting behavior occurred, which might affect the interpretation of the results on condom use consistency. Additionally, data on viral load and antiretroviral regimen were not obtained in the present study. Given the strong evidence showing that PLHIV who are on antiretroviral therapy and maintain a suppressed viral load cannot sexually transmit HIV to their partners [66], future research on condom use of PLHIV should always account for the effect of viral load. Finally, although SEM provides a more nuanced analytic approach to testing a syndemic framework compared with Poisson regression, our SEM model did not directly test for the interactions between component syndemic factors, which are a concept underpinning the theory [15]. Future studies can further advance the methodological basis of syndemic analysis by adopting an interactive approach to understand how different psychosocial conditions interact in ways that synergistically magnify sexual health risks [18].

## 5. Conclusions

This study provides evidence of the associations between HIV-related discrimination, psychosocial syndemics, and condomless sex among PLHIV in Hong Kong. Findings point to the need to develop psychosocial interventions to address internalized stigma and emotional distress for PLHIV in Hong Kong, as a strategy to improve their wellness and reduce continued HIV-related risk behavior. At the structural level, HIV stigma reduction campaigns are necessary in Hong Kong to educate the general public as well as those working in health care settings about HIV transmission and to reduce negative attitudes and judgment toward PLHIV. Moreover, peer-based interventions in Hong Kong might be especially useful to provide social support to PLHIV, as well as to shift social norms toward visibility and inclusion of PLHIV in the society.

## Figures and Tables

**Table 1:** Demographics of the participants (*N* = 291).

Demographic Characteristics	*n* (%)/M (SD)
Gender	
Male	277 (95.8%)
Female	12 (4.2%)
Age	41.8 years (11.1 years)
Ethnicity	
Chinese	271 (94.8%)
South Asian	6 (2.1%)
Caucasian	6 (2.1%)
Others	3 (1.0%)
Education	
Secondary education or less	138 (48.3%)
Tertiary education	133 (46.5%)
Employment status	
Full-time employment	183 (63.3%)
Part-time or irregular employment	46 (15.9%)
Unemployed	36 (12.5%)
Others	24 (8.3%)
Marital status	
Single	194 (67.4%)
Cohabiting/Married	76 (26.4%)
Separated/Divorced/Widowed	20 (6.9%)
Sexual orientation	
Heterosexual	68 (23.7%)
Gay	141 (49.1%)
Bisexual	70 (24.4%)
Questioning	8 (2.8%)
Length of being diagnosed with HIV infection	5.25 years (4.70 years)
Use of antiretroviral therapy	
Yes	254 (90.1%)
No	28 (9.9%)
Number of psychosocial syndemic problem(s)	
0	86 (29.6%)
1	126 (43.3%)
2	54 (18.6%)
3	21 (7.2%)
4	4 (1.4%)
Condom use with steady partners	
Consistent condom use	28 (25.9%)
Inconsistent condom use	80 (74.1%)
Condom use with casual partners	
Consistent condom use	20 (18.7%)
Inconsistent condom use	87 (81.3%)

Cell counts may not add up to total number of participants due to missing or N/A responses.

**Table 2:** Descriptive statistics of and relationships between HIV-related discrimination and psychosocial syndemics.

Variable	All Participants (*N* = 291)				Had HIV-Related Discrimination (*n* = 94)	Did not Have HIV-Related Discrimination (*n* = 197)	Independent Samples *t*-Test		Pearson Correlation Coefficients			
	Mean	SD	Skewness	Kurtosis	Mean (SD)	Mean (SD)	*t*-Value	Cohen’s d	1	2	3	4
1. Depression (1–6)	2.41	0.90	0.79	1.34	2.70 (0.95)	2.28 (0.84)	3.81 **	0.47	-			
2. Anxiety (1–6)	2.57	0.87	0.37	−0.06	2.76 (0.82)	2.49 (0.88)	2.50 *	0.32	0.78 **	-		
3. Social isolation (1–7)	3.27	1.08	0.52	0.36	3.57 (1.14)	3.12 (1.02)	3.39 **	0.42	0.31 **	0.32 **	-	
4. Self-stigma (1–6)	3.82	1.16	−0.34	−0.53	4.00 (1.19)	3.74 (1.14)	1.80	0.22	0.36 **	0.35 **	0.31 **	-

Higher scores indicate higher levels of psychosocial syndemic problems; * *p* < 0.05, ** *p* < 0.01; Cohen’s *d* ≈ 0.20 indicates a small effect size, ≈ 0.50 indicates a medium effect size, and ≈ 0.80 indicates a large effect size.

**Table 3:** Results of Poisson/logistic regressions predicting psychosocial syndemics and sexual risk behavior.

Variable	Psychosocial Syndemics (*N* = 291)		Inconsistent Condom Use with Steady Partners (*n* = 108)		Inconsistent Condom Use with Casual Partners (*n* = 107)	
	IRR	(95% CI)	AOR	(95% CI)	AOR	(95% CI)
HIV-related discrimination						
Yes	1.47 **	(1.14, 1.89)	5.40 *	(1.09, 26.70)	3.24	(0.70, 15.00)
No (reference category)	1.00		1.00		1.00	
Psychosocial syndemics	-	-	3.09 *	(1.22, 7.82)	0.95	(0.48, 1.90)
Gender						
Male	1.73	(0.79, 3.78)	14.54	(0.40, 524.99)	-	-
Female (reference category)	1.00		1.00		-	-
Age	1.00	(0.99, 1.01)	0.98	(0.89, 1.07)	1.05	(0.97, 1.13)
Ethnicity						
Chinese	1.05	(0.58, 1.89)	0.32	(0.02, 4.74)	-	-
Ethnic minority (reference category)	1.00		1.00		-	-
Education						
Secondary education or less (reference category)	1.00		1.00		1.00	
Tertiary education	0.76	(0.58, 1.00)	2.62	(0.52, 13.20)	0.99	(0.23, 4.36)
Employment status						
Full-/part-time employment	0.94	(0.69, 1.27)	0.30	(0.05, 1.91)	1.90	(0.37, 9.68)
Not in employment	1.00		1.00		1.00	
Marital status						
Single (reference category)	1.00		1.00		1.00	
Cohabiting/Married	0.88	(0.64, 1.22)	0.11 *	(0.02, 0.58)	0.24 *	(0.06, 0.99)
Separated/Divorced/Widowed	0.98	(0.57, 1.69)	0.09	(0.01, 1.19)	-	-
Sexual orientation						
Heterosexual	1.06	(0.76, 1.50)	0.13 *	(0.02, 0.94)	1.33	(.13, 13.79)
Sexual minority (reference category)	1.00		1.00		1.00	
Length of being diagnosed with HIV infection	1.00	(1.00, 1.00)	1.00	(0.99, 1.02)	0.98 *	(0.97, 1.00)
Antiretroviral medication use						
Yes	1.37	(0.85, 2.20)	3.98	(0.38, 41.80)	1.61	(0.14, 18.41)
No (reference category)	1.00		1.00		1.00	

IRR = incidence rate ratio; AOR = adjusted odds ratio; CI = confidence interval; * *p* < 0.05, ** *p* < 0.01.
